# Host Preferences Shown by Drosophilids (Diptera) in a Commercial Fruit and Vegetable Distribution Center Follow the Wild Neotropical Pattern

**DOI:** 10.3390/insects14040375

**Published:** 2023-04-11

**Authors:** Laís Barbosa Ribeiro, Carolyn Elinore Barnes Proença, Rosana Tidon

**Affiliations:** 1 Graduate Program in Ecology, Institute of Biological Sciences, University of Brasilia, Brasilia 70910-900, Brazil; laisribeiro015@hotmail.com; 2 Department of Botany, Institute of Biological Sciences, University of Brasilia, Brasilia 70910-900, Brazil; carolyn.proenca@gmail.com; 3 Department of Genetics and Morphology, Institute of Biological Sciences, University of Brasília, Brasilia 70910-900, Brazil

**Keywords:** breeding site, *Drosophila*, fruit markets, invasive species, niche breath, urban ecology, vegetable markets, *Zaprionus*

## Abstract

**Simple Summary:**

Drosophilids (fruit flies) are known as study models in several areas of science. Several drosophilid species have recently attracted public attention because they are expanding their geographic distribution and infesting fruit crops. Here, we investigated the relationship between plants and fruit flies in a commercial fruit and vegetable distribution center in Brazil. We accomplished this by collecting 99,478 kg of potential fruit and vegetable hosts from two time periods separated by a decade, representing 48 plant taxa. The 48,894 fruit flies that emerged from these hosts were identified and attributed to 16 fly species. On both collecting occasions, fruit fly assemblages were strongly dominated by basically the same exotic species, which explore a broader range of hosts, especially those of exotic origin, when compared to native neotropical fruit flies. These results are concerning because the studied site, along with other urban markets around the world, might be acting as a source of widespread generalist species that subsequently disperse into surrounding natural vegetation and crops. As these flies are usually superior competitors, they can promote the local extinction of native fruit flies and therefore contribute to the homogenization of fruit fly communities on larger scales. This phenomenon, known as “biotic homogenization” is worrying scientists worldwide.

**Abstract:**

Although drosophilids have been extensively studied in laboratories worldwide, their ecology is still relatively poorly understood. This is unfortunate because some species are currently expanding their geographic distribution and infesting fruit crops. Here, we investigated the relationship between drosophilids and potential plant hosts in a commercial fruit and vegetable distribution center in the Neotropical region. We collected discarded fruits and vegetables from this commercial center during two time periods (2007–2008 and 2017–2018). Resources were weighted and individually monitored in the laboratory. The drosophilids that emerged were identified, and the relationship between them and their resources was explored. From the 99,478 kg of potential hosts collected, we identified 48 plant taxa, from which 48,894 drosophilids of 16 species emerged. On both collecting occasions, drosophilid assemblages were strongly dominated by basically the same exotic species, which explore a broader range of resources, especially those of exotic origin, when compared to neotropical drosophilids. These results are concerning because the studied site, Along with other urban markets around the world, might be acting as sources of generalist widespread species that disperse to surrounding natural vegetation and contribute to biotic homogenization.

## 1. Introduction

The family Drosophilidae includes more than 4600 nominal species [1] that breed preferentially on fermenting substrates such as fruits, flowers, or fungi [2]. While most species are geographically and ecologically restricted, some are generalists and dispersed beyond their native ranges throughout the world [3]. In Brazil, 364 drosophilid species have been recorded, 350 of which are native and 14 of which are exotic to the Neotropical region [4]. Certain exotic species, such as *Drosophila melanogaster* Meigen and *D. simulans* Sturtevant, probably reached Brazil via ships from Africa in the 16th century. Others arrived in the country more recently as a consequence of globalization. From the late 20th century, five new arrivals in the Neotropics were accurately recorded in the earlier stages of invasion: *D. malerkotliana* Parshad and Paika [5], *Zaprionus indianus* Gupta [6], *D. nasuta* Lamb [7], *D. suzukii* Matsumura [8], and *Z. tuberculatus* Malloch [9]. These introductions are especially worrying because some of these species, such as the spotted wing *Drosophila* (*D. suzukii*, see [10,11]) and the African fig fly (*Z. indianus*, see [12,13]), have become invaders and impact agricultural crops.

The establishment of invasive species in new areas also represents an important threat to biodiversity [14]. Widespread species usually present a high climatic tolerance [15] and explore a wider range of resources than narrowly distributed species. As a result, they can outcompete native species. In a comprehensive survey of fruit-breeding drosophilids and their hosts in the Neotropics, Valadão et al. [16] recorded 180 species of plants (representing 50 families) acting as hosts of 100 drosophilid species. These authors also found that exotic drosophilids breed in more plant species and use exotic hosts more frequently than do Neotropical drosophilids. However, Valadão et al. [16] focused primarily on fruits collected near the host plants; fruits from markets and refuse containers were excluded from their analysis. As there is an expressive drosophilid fauna established in urban environments [17,18,19,20,21], it is worth investigating the drosophilid community associated with the resources available in commercial markets.

The Cerrado biome, also known as Brazilian Savanna, spans most of the Central Brazilian highlands [22] and is one of the world’s biodiversity hotspots due to its high level of endemism and habitat loss [23]. It comprises a savanna of variable structure on the well-drained interfluves, with gallery forests or other moist vegetation following the watercourses [24]. The climate in the Cerrado is tropical dry winter Aw in 95% of the biome, according to the Koeppen classification, and the precipitation is highly seasonal and concentrated during the rainy season from October to April. Currently, 125 neotropical and 13 exotic species of drosophilid have been recorded in this biome [25]. The drosophilids established in a protected area in the center of the Cerrado biome and monitored since 1998 seem to respond to climate seasonality, vegetation heterogeneity, disturbance (including the arrival of exotic species), resource availability, and parasitoids [26,27,28,29,30,31,32,33]. Given the degree of knowledge of this system, it is relevant to investigate the entry routes and establishment sites of exotic species. In this context, food supply and distribution centers in urban areas, which receive products not only from all over the country but also from abroad, become important places to be explored.

The objective of our study was to investigate the relationship between drosophilids and plant species in a distribution center that supplies many urban markets located in the core area of the Cerrado biome. Our main questions were the following: Does the drosophilid community change over time? How are drosophilid species distributed among plant species? Do exotic drosophilids explore a wider range of resources than neotropical drosophilids?

## 2. Materials and Methods

### 2.1. Collections and Taxonomic Determination

Plant resources were collected at the *Centrais de Abastecimento do Distrito Federal* (“Federal District Supply Center” hereafter CEASA-DF), located in the Industry and Supply Sector of Brasília, Brazil. The horticultural products that arrive at CEASA-DF come from different regions of the country and undergo a selection process before being sold. In this process, fruits and vegetables that are deemed unfit for consumption are discarded on the ground, under unloading trucks, and in refuse containers. The collections focused on these decomposing plant resources, which serve as breeding sites for flies and were concentrated over two periods. First, six monthly collections were carried out between August 2007 and January 2008. In the second period, five collections were carried out between October 2018 and May 2019. The collection method in both periods was similar: two collectors randomly collected plant resources. However, in the first period, the collectors spent up to two hours on each collection, while in the second, they spent up to one hour, or until they completed a box of approximately 50 L. The sample units collected (fruits, vegetables, or their fragments) were individually packed and transported to the laboratory.

In the laboratory, each plant sample unit was identified to species (or variety for *Brassica olearacea* L. and *Prunus persica* L.) and classified into types: DF (dry fruits), FF (fleshy fruits), SB (stem bulbs with cataphylls), ST (stem tubers), RT (root tubers), and VL (vegetative leaves). Sample units were then weighed and placed in a transparent plastic container to allow visualization of the hatched flies. In the containers, a thin layer of vermiculite was placed at the bottom to control humidity, and a thin cloth was placed at the opening to trap flies and allow gas exchange. The containers, kept at 25 °C and 12 h:12 h (L:D), were observed every two days. Hatched flies were aspirated and identified by external morphology [34,35] or male terminalia [36,37]. Taxon circumscriptions, names, authors, and geographic distributions of plant and drosophilid species are cited in Valadão et al. [16]. Taxa not included in their study were checked in Taxodros [1] and The World Flora Online [38] for drosophilids and plants, respectively.

### 2.2. Data Analyses

To assess sampling effort and compare species richness for both collection periods [39], we plotted the drosophilid species accumulation curves using a sample-based rarefaction method (plant taxa) using the function “specaccum” in the Vegan package [40] available in R 4.2.2. We used the Whittaker plot to show the species abundance rank and assess the evenness of the community [41]. To calculate the relative abundance of exotic/neotropical species, we added up the abundances of all the species in each category and divided them by the total abundance. We also assessed the drosophilid density (Nflies/g) per plant species; plant samples without emergencies were not considered.

For each collection period, we built matrices of interactions between drosophilid species and plant species, including all recorded interactions. Then, we generate two bipartite networks to visualize the webs. Moreover, we calculated the Spearman correlation between the mass of each vegetable species and the richness and abundance of flies to understand whether the number of interactions reflected the number of resources. For that, the bipartite package [42] and the function “cor.test,” available in R 4.2.2, were used.

The classification of drosophilids as generalists or specialists was based on the criteria established by Magnacca et al. [43]. A species was considered a specialist if two conditions were satisfied: (i) at least two-thirds of its breeding records are associated with a single plant family; and (ii) any other family has <25% of the remaining records, ensuring a clear preference for a single family. For example, a species with 60 breeding records would be considered a specialist if at least 40 records were made in a single plant family and any other family had no more than 15 records. Thus, a drosophilid may be considered a specialist even if it uses alternative plant families as secondary or occasional hosts.

To investigate whether exotic drosophilids explore a wider range of resources than neotropical drosophilids, we calculated the proportion of positive associations observed between Neotropical (N) and exotic (E) hosts (H) and drosophilid (D) species for the four possible pairs: NH × ND, NH × ED, EH × ND, and EH × ED. The expected percentage of associations for each pair was predicted based on the total number of possible associations in the matrix. The adherence between observed and predicted association percentages in each category was tested using the X2 goodness-of-fit test followed by an exact binomial test for each pair as a post hoc test [44]. For this analysis, we only used nominal species.

## 3. Results

**Figure 1:**
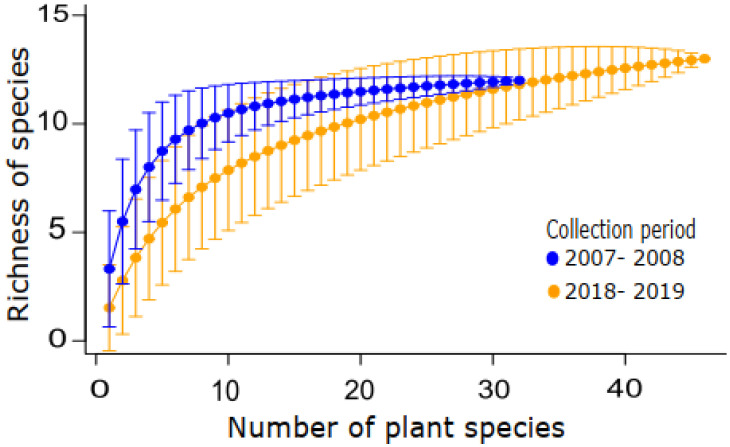
Sample–based rarefaction curves for Drosophilid species were recorded in fruits and vegetables collected in the Federal District Supply Center located in Brasília, Brazil, in two collection periods.

In total, 99.478 kg of plant resources representing 48 species and two varieties (50 taxa in 28 botanical families) were collected and transported to the laboratory (Table 1). From this material, 48,894 drosophilids emerged, representing 16 species (Table 2). Despite the number of sampling units and drosophilids being approximately 50% smaller in the second period compared to the first, the two rarefaction curves stabilized. Thus, in both periods, the sampling effort was sufficient to represent the richness of drosophilids in this urban supply center (Figure 1).

### 3.1. Temporal Changes

**Figure 2:**
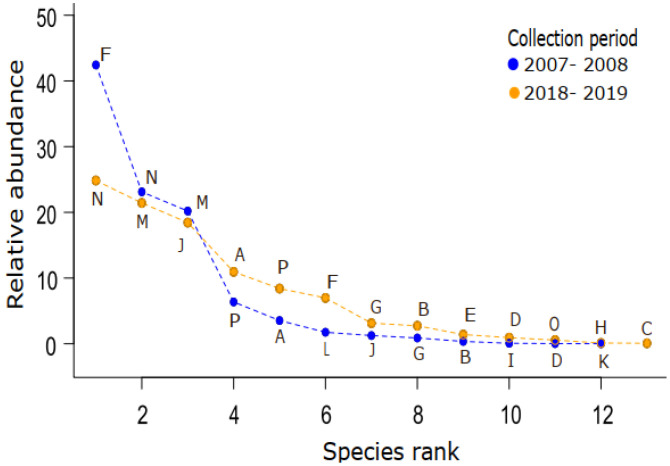
Rank–abundance distribution for drosophilid species recorded in fruits and vegetables collected in the Federal District Supply Center located in Brasília, Brazil, in two collection periods.

The richness of fly species in both periods was similar: 12 and 13 species, with a strong dominance of exotic species (Figure 2). The two most common species present on the two collection occasions (Table 2) corresponded to 98.7% and 93.4% of the total abundance, respectively. The species composition varied: nine occurred in both periods, three occurred exclusively in 2007–2008, and four occurred only in 2018–2019. The plant species most used by flies in 2007–2008 were pumpkin (1.81 flies/g of resource), melon (1.65 flies/g), and pineapple (1.26 flies/g). In the second period (2018–2019), the plant species with the highest density of drosophilids were potato (4.12 flies/g), mango (1.90 flies/g), and pineapple (1.34 flies/g). Several plant taxa did not register drosophilid emergence (Table 1).

### 3.2. Relationships between Plant and Drosophilid Species

**Figure 3:**
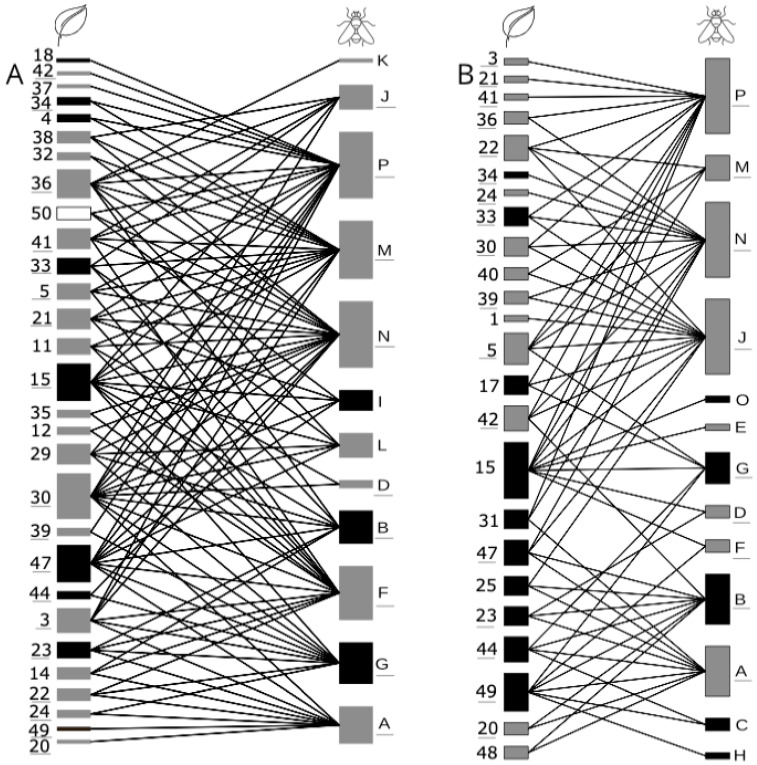
Quantitative food webs for drosophilid species were recorded in fruits and vegetables collected in the Federal District Supply Center located in Brasília, Brazil. (**A**) collection periods 2007–2008 and (**B**) collection periods 2018–2019. For each web, the left bars represent the host plant and the right bars represent drosophilid species. The black lines represent established interactions between plants and drosophilids. The black bars represent native species for the Neotropical region, and the grey bars represent exotic species. The plant and fly species codes are given in Table 1 and Table 2, respectively. Underlined codes represent species occurring in both periods, A and B.

The associations between plant and drosophilid species are shown in Figure 3. The richness of drosophilids in the same plant species varied between 1 and 12, and the most commonly used hosts were pineapple (12 species), banana (11 species), tomato (10 species), melon, and plum (7 species each). Similarly, the number of hosts used by the same species of drosophilid varied between 1 and 22 (Table 2). The drosophilids recorded in most plant species were *Drosophila simulans* (22) and *Zaprionus indianus* (20). However, most species of drosophilids were considered generalists when analyzed using the Magnacca criterion. The exceptions were *D. cardinoides*, *D. nasuta*, *D. repleta*, *D. kikkawai*, and *D. sturtevanti*. The total success rate of fly emergence across the years was 70% of resources (N = 50 species). Fleshy fruits had a success rate of 76.9%; other classes of resources (pooled), such as tubers, bulbs, fruits that are dry at maturity, and leaves, had a lower rate of fly emergence (45.5%).

The correlation between the drosophilid abundance hatched from each plant species and its mass (weighed in the laboratory) was 0.78 (*p* < 0.01) and 0.76 (*p* < 0.01) in the two collection periods, respectively. The correlation between the drosophilid richness hatched from each plant species and its mass was 0.8 (*p* < 0.01) and 0.72 (*p* < 0.01).

### 3.3. Neotropical and Exotic Resources Explored by Neotropical and Exotic Drosophilids

Regarding the geographic (native) origin of flies and host species, the matrix between nominal drosophilids and plants showed that 18.11% of all possible associations were recorded. Although the overall chi-square was not significant (𝜒^2^ = 7.311, d.f. = 3, *p* > 0.05), the use of exotic hosts was significantly lower than expected for neotropical drosophilids and higher than expected for exotic drosophilids (Table 3). The use of neotropical hosts by neotropical and exotic drosophilids follows the same pattern, except that the *p*-value (*p* < 0.07) was marginally significant.

## 4. Discussion

This is the first study to investigate the drosophilid community’s association with resources available in a food supply distribution center in the Neotropics. This study demonstrated that the drosophilid communities sampled in the two periods, separated by a decade, were remarkably similar and were mainly composed of generalist species, with most of them being exotic to the Neotropical region. Exotic drosophilid species clearly explored more hosts than their neotropical counterparts, especially exotic host species, supporting the pattern found for wild drosophilids and their fruit hosts [16].

### 4.1. The Fly Community Remained Relatively Stable after Ten Years

Over time, many similarities have been identified between the two widely isolated samples. Species richness was very similar, and the composition followed the pattern found in natural environments [45]: a few dominant species, generally exotic, in contrast to much more numerous rare species. The relative abundance of species in the community seems to be a predictor of their persistence in the community since the less frequent species (>1.23%) fluctuated the most between the two periods. Our results also support the hypotheses that anthropic environments are favorable for establishing generalist drosophilids [17,19,46]. Markets, especially, provide large amounts and a variety of food resources for drosophilids and are less subject to climate seasonality that affects arthropod communities in tropical savannahs [47,48,49]. Therefore, distribution centers such as the one studied here may function as reservoirs that support generalist and exotic drosophilid populations.

### 4.2. Drosophilid Species Are Not Randomly Distributed among Plant Species

The abundance of drosophilids in each plant species was strongly correlated with the mass brought to the laboratory. Previous studies suggest that resource availability is an important predictor of population growth rates [32,50]. However, fly density fluctuated strongly among different hosts, indicating that plant identity also plays an important role in drosophilid abundance. In drosophilids, there are guilds of species associated with flowers, fungi, and fruits [2,51]. Even within the same guild, however, some host species seem to be especially attractive to flies. For the community studied here, pineapple represents an important resource because it is abundant, supports a high density and richness of drosophilids, and all the collected fragments were colonized. The richness and abundance of drosophilids in each host species probably reflect characteristics such as host chemistry, microbial composition, texture, temperature, and the presence or absence of larvae [52]. *Drosophila* females usually explore the substrate with their proboscis and ovipositor to evaluate its quality for oviposition [53]. As the internal microbiome of a single fly represents a highly reduced subset of the external microbial community, the flies might have some level of control over the yeasts and bacteria that inhabit their digestive tracts [54,55].

As might be expected [2], the emergence of Drosophilids from fleshy fruit (FF) resources was higher than from other types of resources, i.e., bulbs (SB), tubers (RT and ST), leaves (VL), and dry fruits (DF). No emergence of flies was detected from any of the root tubers (RT; N = 4), i.e., beetroot, carrot, Peruvian parsnip, or sweet potato; in nature, such resources would rot underground. There was also no emergence from Okra, commercialized as fruit at a very immature stage, that would have naturally matured into a dry, capsular fruit (DF). Stem and leaf resources (SB, ST, and VL), i.e., cabbage, collard greens, lettuce, onions, potato, and taro had varying levels of emergence. Seven species of *Drosophila* emerged in total from these resources, all of which, except for *D. immigrans*, were among the most abundant species in our dataset, with the three most abundant species of flies, *D. hydei*, *D. simulans,* and *D. melanogaster*, respectively colonizing four, three, or two species of these classes of resources.

The insect’s choice of the host seems to be based on a decision rule that maximizes the expected production of offspring. Polyphagy is putatively selected for when the chances of an organism finding its preferred resource as well as encountering impalatable, toxic, or poor-quality resources are low, while monophagy is selected for when the chances of it finding its preferred resource, as well as encountering impalatable, toxic, or poor-quality resources are high; both strategies aim at maximizing intake of high-quality resources and avoiding a poor-quality diet [56]. The community studied here is mostly composed of generalist species that oviposit in hosts presenting a wide range of conditions. As urban markets tend to maintain high resource availability throughout the year, even if their composition varies according to seasonality, generalist drosophilids can easily find suitable breeding sites and therefore maintain larger populations in these locations. At the other extreme, four species (*D. kikkawai*, *D. repleta*, *D. sturtevanti,* and *D. nasuta*) were bred from a single host and presented relative abundances lower than 0.05%. Although resource fidelity has been described in drosophilid species [57,58], this is not the case here. *D. kikkawai*, *D. repleta*, and *D. sturtevanti* have been recorded in at least six plant families, and *D. nasuta* was a recent invader in the Neotropics during the period of this study; currently, it is widely established in South America, but its breeding sites are virtually unknown [59].

### 4.3. Exotic Drosophilids Use More Resources Than Neotropical Drosophilids

The three drosophilid species that exploit the most resources—*Drosophila simulans*, *Zaprionus indianus*, and *D. melanogaster*—are endemic to Africa [3,35], widely distributed throughout the world [1], and abundant in South America [26,32,60]. The success of exotic drosophilids partly stems from their ability to exploit a wider range of plant species. This finding supports Valadão et al. [16], who suggest three complementary hypotheses to explain the broader resource richness used by exotic drosophilids. First, exotic species have survived the trials of introduction, establishment, and dispersion in a new area; thus, they should be adapted to an array of conditions. Second, they may favor their own offspring by inoculating microbes at their breeding sites through their fecal deposits and oviposited eggs [61,62], enhancing the resources available to hatching larvae. Finally, exotic drosophilids can be superior competitors compared to native species: *D. melanogaster* can affect the size of other species when sharing the same resource patch during the larval stage [63], and *Z. indianus* can chase away other species of drosophilids that leave potential breeding sites without laying eggs (see video S1 in Valadão et al. [16]). These processes can promote niche breadth expansion via adaptive evolution [64].

Exotic drosophilids were particularly successful in breeding on exotic host plants, which usually represent much of the resources available in fruit and vegetable distribution centers. Consequently, these markets could provide plentiful breeding sites that act as sources of species that colonize surrounding patches of natural vegetation as well as a possible selection for enhanced generalism. The dispersion of competitive species from urban markets to nature, in turn, could contribute to biotic homogenization by eliminating native species [65,66]. The decline of insect populations and species around the world has been intensely debated, and while there are studies suggesting dramatic rates of extinction of insect species over the next few decades [67,68], there is also some criticism of the methods used to estimate this decline [69].

### 4.4. Future Research

Drosophilids are good models in many areas of research [70], including conservation and invasion biology [71,72]. In addition to the high-quality, extensively researched publications about nearly all aspects of these flies, there are also complete databanks dealing with *Drosophila* genetics (https://flybase.org/, accessed on 18 January 2023) and taxonomy, including geographical distribution (https://www.taxodros.uzh.ch/, accessed on 18 January 2023). An interesting line of research would be testing the hypothesis that geographically widespread generalists have an apparently greater tendency to use novel, exotic hosts than geographically constrained specialists, as found for butterflies [73,74]. Another promising avenue for research is the standardized approach for systematically monitoring alien species and tracking biological invasions [75]. Considering that exotic species can interact, favoring each other and establishing new ones [76], monitoring becomes especially relevant. In the interval between the two collection periods, the establishment of two exotic species in the Neotropical region was recorded: *Drosophila nasuta* and *D. suzukii*. The first, *D. nasuta*, was registered by us in the second collection period as a rare specialist species (<0.5% of the sample came from a single host), although it was much more abundant (211 records) than the other newly–recorded species of the second period combined (113). This result certainly reflects the short time of introduction at the time of collection, since there are records of *D. nasuta* associated with different trophic levels [77]. Furthermore, given its distribution potential in different Neotropical biomes [78,79], *D. nasuta* possibly uses a variety of resources that should mediate its dispersal. The second, *D. suzukii*, was not found in our samples, although it occurs in natural environments adjacent to the study area [80]. Known as the spotted-winged *Drosophila*, *D. suzukii* has already been found on 64 host plants in 25 families in Latin America, most of which are exotic species [10]. This is an important worldwide pest that infests wild and cultivated small soft-skinned fruits [81], and the rarity of this type of host in our samples might explain its absence in the present study.

## 5. Conclusions

In short, our study suggests that the drosophilid community established in a fruit and vegetable distribution center located in the core area of South America is relatively stable and dominated by generalist exotic species. Neotropical species were also present, but in general, they were rarer and showed a narrower niche breath. These results are worrying because the studied site, along with other urban markets around the world, might be acting as sources of generalist widespread species that disperse to surrounding natural vegetation and contribute to biotic homogenization.

## Figures and Tables

**Table 1:** Plant families and taxa collected in the Federal District Supply Center in Brasília, Brazil (CEASA).

Family	Taxa	Popular Name	Code	Type	Mass (g)	Empty Mass (%)	Collection Period
Actinidiaceae	*Actinidia chinensis* Planch. ^E^	Kiwi; Kiwi	1	FF	592.4	86.26	1′, 2
Amaranthaceae	*Beta vulgaris* L. ^E^	Beterraba; Beetroot	2	**RT**	482.9	100	2′
Amaryllidaceae	*Allium cepa* L. ^E^	Cebola; Onion	3	**SB**	3037.1	57.81	1, 2
Anacardiaceae	*Anacardium occidentale* L. ^N^	Caju; Cashew fruit	4	FF	524.0	25.38	1
	*Mangifera indica* L. ^E^	Manga; Mango	5	FF	8595.8	69.42	1, 2
	*Spondias mombin* L. ^N^	Cajá,Cajazinho; Java plum	6	FF	107.2	100	2′
	*Spondias purpurea* L. ^N^	Ciriguela, Seriguela; Gambia plum, Purple mombin	7	FF	8.9	100	2′
Annonaceae	*Annona squamosa* L. ^N^	Pinha, Fruta do Conde; Custard apple	8	FF	398.6	100	2′
Apiaceae	*Arracacia xanthorrhiza* Bancr. ^N^	Batata baroa, Mandioquinha; Arracache, Peruvian parsnip	9	**RT**	79.7	100	2′
	*Daucus carota* L. ^E^	Cenoura; Carrot	10	**RT**	501.8	100	2′
Araceae	*Colocasia esculenta* (L.) Schott ^E^	Inhame; Cocoyam, Taro	11	**ST**	711	73.42	1
Asteraceae	*Lactuca sativa* L. ^E^	Alface; Lettuce	12	**VL**	290	0	1
Brassicaceae	*Brassica oleracea* L. var. *acephala* DC. ^E^	Couve; Collard greens, Kale	13	**VL**	59.9	100	2′
	*Brassica oleracea* L. var. *capitata* L. ^E^	Repolho; Cabbage	14	**VL**	585	0	1
Bromeliaceae	*Ananas comosus* (L.) Merr. ^N^	Abacaxi; Pineapple	15	FF	27,195.5	0	1, 2
Cactaceae	*Selenicereus undatus* (Haw.) D.R.Hunt ^N^	Pitaya; Dragon fruit	16	FF	205.9	100	2′
Caricaceae	*Carica papaya* L. ^N^	Mamão; Papaya	17	FF	6256.2	87.24	1′, 2
Caryocaraceae	*Caryocar brasiliense* Cambess. ^N^	Pequi; no English name	18	FF	171	17.54	1
Convolvulaceae	*Ipomoea batatas* (L.) Lam. ^N^	Batata doce; Sweet potato	19	**RT**	125.2	100	2′
Cucurbitaceae	*Cucumis anguria* L. ^E^	Maxixe; West indian gherkin	20	FF	577.7	78.38	1, 2
	*Citrullus lanatus* (Thunb.) Matsum. and Nakai^E^	Melancia; Watermelon	21	FF	6071.9	2.86	1, 2
	*Cucumis melo* L. ^E^	Melão; Melon	22	FF	962.9	0	1, 2
	*Curcubita moschata* Duchesne ^N^	Abóbora; Pumpkin, Winter squash	23	FF	1497.3	11.98	1, 2
	*Cucumis sativus* L. ^E^	Pepino; Cucumber	24	FF	844.7	58.01	1, 2
	*Sicyos edulis* Jacq. ^N^	Chuchu; Chayote, Corstophine	25	FF	558.9	31.10	2
Ebenaceae	*Diospyros kaki* L.f. ^E^	Caqui; Persimmon	26	FF	282.8	100	2′
Lauraceae	*Persea americana* Mill. ^N^	Abacate; Avocado	27	FF	1201.4	100	2′
Malvaceae	*Hibiscus esculentus* L. ^N^	Quiabo; Okra, Gumbo, Lady’s fingers	28	**DF**	62.9	100	2′
Moraceae	*Artocarpus heterophyllus* Lam. ^E^	Jaca; Jackfruit	29	FF	1490	0	1
Musaceae	*Musa* x paradisiaca L. ^E^	Banana; Banana	30	FF	5860.3	34.55	1, 2
Myrtaceae	*Psidium guajava* L. ^N^	Goiaba; Guava	31	FF	1035.7	54.28	1′, 2
Oxalidaceae	*Averrhoa carambola* L. ^E^	Carambola; Star fruit	32	FF	105.3	13.58	1, 2′
Passifloraceae	*Passiflora edulis* Sims ^N^	Maracujá; Passion fruit	33	FF	1800.9	43.17	1, 2
Rosaceae	*Fragaria vesca* L. ^N^	Morango; Strawberry	34	FF	209.8	64.59	1, 2
	*Malus domestica* (Suckow) Borkh. ^E^	Maçã; Apple	35	FF	5128	94.,44	1, 2′
	*Prunus domestica* L. ^E^	Ameixa; Plum	36	FF	1393	63.03	1, 2
	*Prunus persica* (L.) Batsch ^E^	Pêssego; Peach	37	FF	709	56.56	1, 2′
	*Prunus persica* var. *nucipersica* (L.)C.K. Schneid. ^E^	Nectarina; Nectarine	38	FF	433.8	76.26	1, 2′
	*Pyrus communis* L. ^E^	Pera; Pear	39	FF	1568.4	73.64	1, 2
Rutaceae	*Citrus x aurantiifolia* (Christm.) Swingle ^E^	Limão; Lime	40	FF	571	80,14	2
	*Citrus* x *reticulata* Blanco ^E^	Mexirica, Bergamota; Tangerine	41	FF	2391	38,62	1, 2
	*Citrus sinensis* (L.) Osbeck ^E^	Laranja; Orange	42	FF	2681.2	47.,78	1, 2
Sapindaceae	*Litchi chinensis* Sonn. ^E^	Lichia; Lychee	43	FF	19.9	100	2′
Solanaceae	*Capsicum annuum* L. ^N^	Pimentão; Bell pepper	44	FF	2346.5	71.02	1, 2
	*Capsicum chinense* L. ^N^	Pimenta; Chili pepper	45	FF	25.5	100	2′
	*Solanum aethiopicum* L. ^E^	Jiló; Bitterberry	46	FF	239.6	100	2′
	*Solanum lycopersicum* Lam. *^N^*	Tomate; Tomato	47	FF	5592	39.33	1, 2
	*Solanum melongena* L. ^E^	Beringela; Eggplant, Aubergine	48	FF	360.5	39.92	2
	*Solanum tuberosum* L. ^N^	Batata; Potato	49	**ST**	3420.4	79.69	1, 2
Vitaceae	*Vitis vinifera* L. × *Vitis labrusca* ^EN^	Uva; Grape	50	FF	108.4	40.96	1, 2′

^E^: exotic; ^N^: neotropical. Popular names: Brazilian Portuguese; English names. DF: dry fruit; FF: fleshy fruit; RT: root tuber; SB: stem bulb with cataphylls; ST: stem tuber; VL: vegetative leaf. Resources that are not FF in bold. Code: as in Figure 3. Mass: total collected. Empty mass: mass without the emergence of drosophilids. Collection periods 1: 2007–2008; 2: 2018–2019; apostrophe (′): there was no emergence of flies.

**Table 2:** Genera, subgenera, groups, and species of Drosophilidae recorded on fruits and vegetables collected in the Federal District Supply Center located in Brasília, Brazil, in two collection periods: 2007–2008 (1) and 2018–2019 (2). ^E^: exotic; ^N^: neotropical; Plant Fam/Spp: number of plant families and plant species with drosophilid records.

Genus	Subgenus	Group	Species	Code	Plant Fam/Spp	Abundance	
						2007–2008	2018–2019
*Drosophila*	*Dorsilopha*	*busckii*	*D. busckii* Coquillett ^E^	A	6/13	1198	1647
	*Drosophila*	*cardini*	*D. cardini* Sturtevant ^N^	B	9/14	121	412
			*D. cardinoides* Dobzhansky and Pavan ^N^	C	1/2	0	11
		*immigrans*	*D. immigrans* Sturtevant ^E^	D	4/4	8	140
			*D. nasuta* Lamb ^E^	E	1/1	0	211
		*repleta*	*D. hydei* Sturtevant ^E^	F	11/14	14,361	1049
			*D. mercatorum* Patterson and Wheeler ^N^	G	9/13	299	472
			*D. repleta* Wollaston ^N^	H	1/1	0	17
		*willistoni*	*D. nebulosa* Sturtevant ^N^	I	4/4	25	0
	*Sophophora*	*melanogaster*	*D. ananassae* Doleschall ^E^	J	10/14	424	2775
			*D. kikkawai* Burla ^E^	K	1/1	2	0
			*D. malerkotliana* Parshad and Paika ^E^	L	6/6	592	0
			*D. melanogaster* Meigen ^E^	M	11/15	6838	3225
			*D. simulans* Sturtevant ^E^	N	15/22	7824	3739
		*saltans*	*D. sturtevanti* Duda ^N^	O	1/1	0	85
*Zaprionus*			*Z. indianus* Gupta ^E^	P	14/20	2154	1265

**Table 3:** Percentages of observed interactions (matrix cell occupancy) between drosophilids and host species (i.e., 100% would mean all possible drosophilids using all possible hosts) for each of four possible classes of interaction: (i) Neotropical drosophilid × Neotropical host; (ii) Neotropical drosophilid × exotic host; (iii) exotic drosophilid × Neotropical host; and (iv) exotic drosophilid × exotic host. *p*-values refer to pair Binomial tests.

	Plant Hosts			
Drosophilids	Neotropical (21 Taxa)		Exotic (29 Taxa)	
Neotropical (6 species)	19/126 = 15.08%	(*p* = 0.067)	15/174 = 8.93%	(*p* < 0.001)
Exotic (10 species)	40/210 = 19.05%	(*p* = 0.064)	68/280 = 24.28%	(*p* = 0.012)

## Data Availability

The data presented in this study are openly available in this manuscript.
